# TKI Use and Treatment-Free Remission in Chronic Myeloid Leukemia: Evidence from a Regional Cohort Study in the Canary Islands

**DOI:** 10.3390/hematolrep17040039

**Published:** 2025-08-04

**Authors:** Santiago Sánchez-Sosa, Ruth Stuckey, Adrián Segura Díaz, José David González San Miguel, Ylenia Morales Ruiz, Sunil Lakhawani Lakhawani, Jose María Raya Sánchez, Melania Moreno Vega, María Tapia Torres, Pilar López-Coronado, María de las Nieves Saez Perdomo, Marta Fernández, Cornelia Stoica, Cristina Bilbao Sieyro, María Teresa Gómez Casares

**Affiliations:** 1 Hospital Universitario de Gran Canaria Doctor Negrin (HUGCDN), 35019 Las Palmas de Gran Canaria, Spain; 2 Department of Specific Didactics, Faculty of Education, Universidad de Las Palmas de Gran Canaria (ULPGC), 35004 Las Palmas de Gran Canaria, Spain; 3 Instituto de Investigación Sanitaria de Canarias (IISC), 35019 Las Palmas de Gran Canaria, Spain; 4 Department of Medical and Surgical Sciences, Faculty of Health Sciences, Universidad de Las Palmas de Gran Canaria (ULPGC), 35016 Las Palmas de Gran Canaria, Spain; 5 Complejo Hospitalario Insular de Gran Canaria Materno Infantil (CHUIMI), 35016 Las Palmas de Gran Canaria, Spain; 6 Complejo Hospitalario Universitario de Canarias (CHUC), 38320 San Cristóbal de La Laguna, Spain; 7 Hospital José Molina Orosa (HJMO), 35500 Arrecife, Spain; 8 Hospital General de La Palma (HGP), 38713 Breña Alta, Spain; 9 Hospital General de Fuerteventura (HGF), 35600 Puerto del Rosario, Spain; 10 Department of Morphology, Faculty of Health Sciences, Universidad de Las Palmas de Gran Canaria (ULPGC), 35016 Las Palmas de Gran Canaria, Spain

**Keywords:** chronic myeloid leukemia (CML), tyrosine kinase inhibitors (TKIs), treatment-free remission (TFR)

## Abstract

**Background/Objectives**: The advent of tyrosine kinase inhibitors (TKIs) revolutionized the management of chronic myeloid leukemia (CML), achieving survival rates near those of the general population. Despite this success, prolonged therapy presents challenges, including physical, emotional, and financial burdens. Treatment-free remission (TFR), defined as sustained deep molecular response (DMR) after discontinuing TKIs, has emerged as a viable clinical goal. This study evaluates real-world data from the Canary Islands Registry of CML (RCLMC) to explore outcomes, predictors, and the feasibility of TFR. **Methods**: This retrospective observational study included 393 patients diagnosed with CML-CP between 2007 and 2023. Molecular response was monitored according to international guidelines. Survival probabilities were estimated using the Kaplan–Meier method. Logistic regression analysis was performed to identify predictors of molecular relapses after TKI discontinuation. **Results**: Of the 383 patients who received TKI treatment, 58.3% achieved molecular response grade 2 (MR2) (BCR-ABL1 ≤ 1%), 95.05% achieved MR2, and 50.5% reached MR4 within the first year. Of the 107 patients attempting TFR, 73.2% maintained remission at 36 months. Relapses occurred in 24 patients, all regaining molecular response upon reintroduction of TKIs. No cases of disease progression were observed. **Conclusions**: Our findings support the feasibility and safety of TFR in a real-world clinical setting for well-selected patients, with outcomes consistent with international studies. The study underscores the importance of molecular monitoring and patient-specific strategies to optimize outcomes.

## 1. Introduction

Chronic myeloid leukemia (CML) has witnessed a revolutionary transformation in its management due to the advent of tyrosine kinase inhibitors (TKIs). These inhibitors target the BCR-ABL1 oncoprotein, a constitutively active tyrosine kinase resulting from the Philadelphia chromosome, which arises from the reciprocal translocation t(9;22)(q34;q11), leading to the formation of the BCR-ABL1 fusion gene. TKIs have dramatically improved patients’ survival rates and quality of life, turning CML into a largely manageable chronic disease. The development of TKIs has also enabled many patients to achieve deep molecular responses (DMRs), a prerequisite for exploring treatment-free remission (TFR), which allows selected patients to discontinue therapy while maintaining disease control [1,2].

Multiple TKIs have been approved for treating CML, categorized into different generations based on their potency and ability to overcome resistance (Table 1):

Nowadays, the concept of TFR has gained significant traction, particularly for patients who achieve sustained DMR while on TKI therapy. The ability to discontinue TKIs not only reduces long-term side effects such as cardiovascular complications and metabolic disturbances but also improves patients’ quality of life by alleviating treatment-related fatigue, gastrointestinal discomfort, and other chronic adverse effects. Additionally, TFR decreases the financial burden of lifelong therapy, offering both economic relief for patients and cost savings for healthcare systems [5]. Despite these advantages, TFR is successful in only about 50% of patients, as molecular relapses often necessitate the resumption of therapy [4,7].

The factors influencing successful TFR include the depth and duration of molecular responses, patient adherence to treatment, and the presence of specific biological markers such as residual leukemic stem cells [8,9]. Emerging evidence highlights that earlier achievement of DMR significantly predicts TFR success, particularly in patients treated with second-generation TKIs [10,11]. Additionally, combining TKIs with immune-modulating therapies, such as interferon-alpha or novel immunotherapies, is being explored as a promising strategy to enhance immune surveillance and improve TFR outcomes.

The objective of the present study is to analyze real-life data from patients with chronic myeloid leukemia included in the Canary Islands Chronic Myeloid Leukemia Registry (RCLMC) and compare these findings with international studies.

## 2. Materials and Methods

Study Design and Data Collection

This study utilized data from the Canary Islands CML Registry (RCLMC), a regional database established in 2007 to track patients diagnosed with CML in the chronic phase (CML-CP). The registry includes comprehensive clinical and molecular data for 393 patients diagnosed between 2007 and 2023. This retrospective observational study focused on the subset of 383 patients treated with TKIs, including imatinib, dasatinib, nilotinib, bosutinib, ponatinib, and asciminib. Patient data were extracted for analysis, including demographics, treatment history, molecular response levels, and outcomes following TKI discontinuation.

TKI discontinuation was considered in patients who had achieved and sustained a deep molecular response (MR4 or deeper) for at least 24 months, in accordance with international recommendations. Eligibility for treatment-free remission was assessed by the treating physician based on molecular response history, adherence, and clinical stability. Molecular relapse during TFR was defined as the confirmed loss of major molecular response (MMR, BCR-ABL1 > 0.1% IS) in two consecutive tests. Patients were monitored monthly during the first year, every two months between months 12 and 24, and every three months thereafter

The data from the RCLMC were integrated into an online platform designed by the investigator group, utilizing the OMOP common data model. This integration facilitates advanced statistical analyses and enhances data interoperability. Furthermore, this approach streamlines data harmonization and enables shared infrastructures, fostering comparisons and collaborations with other registries and expanding the utility of the RCLMC for global research initiatives.

Molecular Monitoring and Definitions

Molecular response was assessed using quantitative polymerase chain reaction (qPCR) to measure BCR-ABL1 transcript levels, following International Scale (IS) standards [12,13]. The following molecular response levels were defined:

- MR3: Major molecular response, BCR-ABL1 ≤ 0.1%.
- MR4: Deep molecular response, BCR-ABL1 ≤ 0.01%.
- MR4.5: Sustained deep molecular response, BCR-ABL1 ≤ 0.0032%.

Molecular relapse during TFR was defined as the loss of MMR in two consecutive samples. Relapsed patients were immediately restarted on TKI therapy and monitored for molecular response recovery.

Treatment and Discontinuation Protocols

Patients were treated with first-, second-, or third-generation TKIs according to standard clinical guidelines and the preferences of the prescribing physician. All TKIs were administered following the standard dosing regimens approved in their respective summary of product characteristics (SPC), consistent with international clinical guidelines. TKI discontinuation was offered to patients achieving and sustaining DMR for at least 24 months. After discontinuation, patients underwent intensive molecular monitoring of BCR-ABL levels using qPCR. Monitoring was performed monthly during the first 12 months, every two months between months 12 and 24, and then every 12 weeks.

Statistical Analysis

Descriptive statistics were used to summarize patient demographics, treatment lines, and molecular response rates. Percentages were calculated using the total number of evaluable patients as denominators in each subgroup. Associations between early molecular response (EMR) and later deep molecular responses (MR4, MR4.5) were evaluated using Pearson’s chi-squared test and simple odds ratios. All statistical calculations were performed manually using Microsoft Excel (Microsoft Corporation, Redmond, WA, USA). A *p* value < 0.05 was considered statistically significant.

## 3. Results

At the current date, the RCLMC includes complete data for 393 CML-CP patients, 209 males and 184 females, with a median age of 56 years at diagnosis (range 18–98 years old). Among these patients, 383 received TKI treatment and are distributed across the different treatment lines as shown in the following table (Table 2):

Of the 383 patients who received first-line TKI treatment, molecular response data was available for 218 patients. Of these, 58.25% of patients achieved a molecular response of less than 1% at 3 months.

Analyzing the treatment lines, the following percentages of patients achieved the following molecular responses (Table 3):

In total, 50.50% of patients in the first-line treatment group achieved a molecular response of MR4 or MR4.5. According to the ELN 2020 recommendations, 79.2% of patients had achieved an optimal response (<0.1%) by 12 months (Table 4, Table 5 and Table 6).

The association between achieving an early molecular response (EMR) and molecular responses (MR4 or MR4.5) at 12, 18, and 24 months with first-line TKI and second-line TKI was analyzed (Table 7 and Table 8):

We observed that achieving an EMR was statistically associated with a deep molecular response to first-line TKIs at 12 months, but not at later time points, whereas it was not associated with a deep molecular response to second-line TKIs after 12, 18, or 24 months.

The average time, measured in months, between initiating second-line and third-line treatment with TKIs in our series is 32.6 months, with a range of 0.42–175.7 months.

### 3.1. Treatment-Free Remission

Among the total series of 383 patients, 27.9% (107 patients) achieved treatment-free remission (TFR), 79.4% received only one line of TKI treatment, while 20.6% received at least two lines of TKI treatment prior to discontinuation. The main cause of stopping treatment was TFR (76.92%), followed by drug intolerance (15.39%).

The TFR rate at 12 months was 76.4% (95% CI: 68.7–84.9%), and it remained at 73.2% at 24 and 36 months. In total, 24 of the 107 patients experienced major molecular response loss, typically within the first few months after discontinuing TKI therapy, although exact timings could not be consistently retrieved due to data limitations inherent in the retrospective study design, and were reintroduced to TKI treatment. Using the Pearson test, we observed a positive correlation between TFR and the number of months in MR4 (*p* = 0.047; Pearson coefficient 0.21).

When focusing on patient safety, none of the patients who discontinued treatment have developed disease progression. All patients regained a molecular response within the first three months after reintroducing medication.

### 3.2. Adherence

Overall, treatment adherence in all treatment lines is good (>80%), especially in second-line and subsequent lines, as healthcare professionals emphasize the importance of adherence during each patient visit, particularly when detecting a loss of response.

## 4. Discussion

### 4.1. Real-World TKI Utilization Patterns

The RCLMC data reflect diverse patterns of TKI use, with first-line imatinib remaining the most commonly prescribed therapy. Approximately 66% of patients in the registry received imatinib as their initial treatment, followed by second-generation TKIs such as dasatinib and nilotinib, which accounted for a combined 30% of first-line treatments. A smaller cohort transitioned to third-generation TKIs, including bosutinib and ponatinib, primarily in cases of resistance or intolerance to earlier lines of therapy [14]. Molecular mutation analysis, such as BCR-ABL1 kinase domain mutation testing, was not systematically conducted in resistant cases across the cohort, and therefore mutation data were not available for inclusion in this study.

Patients achieving molecular response under second-generation TKIs demonstrated faster and deeper reductions in BCR-ABL1 transcript levels compared to those on imatinib. This data aligns with recent findings suggesting that second- and third-generation TKIs offer superior rates of MR4 and MR4.5 [15]. However, the RCLMC also emphasizes that a significant proportion of patients on imatinib achieved sustained DMR, reinforcing its continued relevance as a cost-effective first-line therapy [16].

### 4.2. Treatment-Free Remission

Different clinical trials such as EURO-SKI, DESTINY, ENESTfreedom, and ENESTop have provided significant insights into the critical factors influencing treatment-free remission in chronic myeloid leukemia patients. The EURO-SKI trial emphasized deep molecular response duration as a key predictor for successfully discontinuing tyrosine kinase inhibitors. Patients maintaining a DMR for at least three years prior to treatment cessation showed significantly higher TFR success rates. Furthermore, the overall duration of TKI treatment was a relevant factor, with greater success observed in patients treated for more than six years [17].

The DESTINY trial explored dose-reduction strategies prior to discontinuation. It reported that only 2.4% of patients experienced molecular relapse following a gradual TKI dose reduction. This approach not only minimized associated toxicities but also enhanced TFR success by extending the duration of pre-cessation DMR [18,19].

The ENESTfreedom and ENESTop trials have further strengthened the evidence base for TFR. The ENESTfreedom trial focused on first-line nilotinib treatment and demonstrated that approximately 50% of patients achieved successful TFR at 96 weeks following therapy discontinuation [20]. Similarly, the ENESTop trial, which targeted patients transitioning from imatinib to nilotinib due to suboptimal responses or intolerance, reported durable TFR rates in patients achieving sustained DMR after switching therapies [15]. These studies underscore the benefits of second-generation TKIs, such as nilotinib, in achieving deeper molecular responses more quickly, thereby improving the likelihood of successful TFR.

All studies highlight the importance of stringent and continuous molecular monitoring. As a retrospective observational study, the long follow-up periods of these patients may result in data loss or even complete loss of follow-up for some patients, potentially leading to valuable data being missing.

The findings from the Canary Islands CML Registry (RCLMC) not only highlight the feasibility and safety of treatment-free remission but also offer valuable insights into the real-world utilization of tyrosine kinase inhibitors. The RCLMC’s extensive dataset reveals patterns in TKI use that have significant implications for optimizing patient outcomes, both in achieving deep molecular response and maintaining remission post-discontinuation [9,21].

### 4.3. Implications for TFR Attempts

The study underscores the importance of aligning TKI selection with patient-specific factors. For instance,

- Patients on second-generation TKIs, who typically achieve DMR more rapidly, may require less time in sustained remission before attempting TFR [22];
- In contrast, patients on imatinib may benefit from longer periods of DMR prior to discontinuation to minimize relapse risk discontinuation [9].

Furthermore, the RCLMC data demonstrate the pivotal role of molecular monitoring in guiding TFR. The frequency and precision of BCR-ABL1 transcript measurements directly influenced relapse detection and patient outcomes. This fact underscores the need for robust laboratory infrastructure, which the RCLMC has continuously developed, to ensure accurate and timely molecular assessments.

### 4.4. Broader Clinical Implications

The diverse use of TKIs within the RCLMC reflects real-world challenges, including managing resistance, intolerance, and patient adherence. Approximately 15% of patients required transitions between two or more TKI lines due to suboptimal response or adverse effects [23]. These findings highlight the importance of personalized treatment plans incorporating clinical and psychosocial factors to optimize the use of TKIs and support successful TFR [9,24].

According to the RCLMC data, this study corroborates the growing body of evidence that supports the feasibility and safety of TFR in well-selected CML patients. International trials have consistently demonstrated that TFR is achievable in 40–60% of patients with sustained DMR [10,22]. In the present study, we observed that 73.2% of patients attempting TFR maintained remission at 36 months, surpassing the averages reported in prior research. This underscores the potential for regional and individualized treatment approaches to enhance outcomes.

A critical predictor of successful TFR identified in this study was the achievement of an early molecular response, defined as BCR-ABL1 ≤ 10% at three months. Patients achieving EMR demonstrated a significantly higher likelihood of maintaining MMR and, subsequently, TFR. This finding aligns with the existing literature, highlighting EMR as a cornerstone of effective TKI therapy and a reliable surrogate marker for long-term success [13,25]. Furthermore, sustained MR4 or MR4.5 prior to discontinuation was pivotal, reinforcing international guidelines that advocate for stringent molecular criteria before attempting TFR [16].

Relapse management is a crucial aspect of TFR. In this cohort, all patients who experienced molecular relapse (24 individuals) regained molecular response upon reinitiating TKI therapy, typically within three months. This finding demonstrates that TFR, when appropriately monitored, poses minimal risk of disease progression, provided relapses are promptly identified and managed. The absence of disease progression in this study supports the safety of TFR in a real-world clinical setting, provided robust molecular monitoring systems are in place [10].

From a clinical perspective, the findings also underscore the need to tailor TFR strategies to patient-specific factors. For instance, patients with comorbidities or those experiencing adverse effects from long-term TKI use may benefit more significantly from TFR. Conversely, individuals with a history of suboptimal response to therapy may require additional interventions or prolonged molecular surveillance prior to attempting TFR [24].

Patients often express relief at discontinuing TKI therapy, citing improved quality of life and reduced treatment burdens. However, the anxiety associated with regular molecular testing and potential relapse must be addressed through patient education and supportive care frameworks. Moreover, TFR represents a significant cost-saving measure, reducing long-term healthcare expenditures for patients and systems. The psychological and financial implications of TFR merit further exploration. And future studies should incorporate these dimensions to provide a holistic understanding of TFR’s impact [26].

Our findings are consistent with those reported in key international studies, particularly EURO-SKI [17] and STIM [14], which have investigated treatment-free remission (TFR) in large cohorts of CML patients. In EURO-SKI, approximately 50% of patients maintained molecular remission at 24 months following TKI discontinuation. Similarly, the STIM trial reported that around 40% of patients remained in remission at 24 months. In our cohort, the TFR rate was 73.2% at 36 months, which is comparatively higher. This may reflect a combination of careful patient selection, real-world clinical management, and slightly less stringent eligibility criteria (sustained MR4 for at least 24 months, rather than MR4.5 as required in STIM). Despite these differences, our findings reinforce the safety and feasibility of TFR, with no progression events and full molecular recovery after relapse, in line with these landmark studies.

Finally, the findings from the RCLMC emphasize the importance of regional data in shaping global treatment paradigms. Variations in healthcare infrastructure, patient demographics, and access to molecular diagnostics can influence TFR outcomes. Therefore, initiatives like the RCLMC play a vital role in contextualizing international guidelines to local settings, ensuring equitable and effective care.

## 5. Conclusions

The Canary Islands Registry of CML (RCLMC) provides valuable real-world evidence on the utilization of tyrosine kinase inhibitors in patients with chronic-phase CML. The data emphasize the continued relevance of first-line imatinib as a cost-effective option for achieving molecular response, while second- and third-generation TKIs deliver faster and deeper responses, particularly for high-risk patients or those requiring rapid BCR-ABL1 transcript reduction.

Each generation of TKIs has contributed to the evolving landscape of CML treatment, addressing gaps in resistance and intolerance. The findings highlight the importance of tailored therapeutic approaches and robust molecular monitoring systems to ensure optimal patient outcomes.

Notably, the RCLMC also supports the viability of treatment-free remission in patients who achieve and sustain major molecular response. Its integration of advanced diagnostic technologies ensures precise relapse detection and timely reintroduction of therapy when needed.

By combining regional insights with real-world data, the RCLMC serves as a model for enhancing CML management globally. It underscores the potential to reduce treatment burdens, improve patient quality of life, and optimize healthcare resource utilization through individualized treatment strategies and continuous molecular monitoring.

## Figures and Tables

**Table 1:** An overview of tyrosine kinase inhibitors (TKIs) approved for the treatment of chronic myeloid leukemia (CML), categorized by generation. The classification reflects differences in potency, target specificity, and capacity to overcome resistance-associated BCR-ABL1 mutations.

Generation	Drug	Summary
First	Imatinib	The first TKI approved for CML, which revolutionized treatment by targeting the ATP-binding site of the BCR-ABL1 protein. While effective in many patients, resistance due to mutations in the tyrosine kinase domain of the BCR-ABL1 gene or intolerance to the drug prompted the development of second-generation TKIs [3].
Second	Dasatinib	A dual SRC/ABL inhibitor with higher potency than imatinib. It can overcome several mutations that confer resistance to imatinib, including most mutations except T315I [4].
	Nilotinib	Designed to specifically target BCR-ABL1 with greater selectivity and potency than imatinib, it also provides better efficacy in achieving deep molecular responses [1].
	Bosutinib	The drug offers a broader spectrum of activity by inhibiting ABL1 and SRC family kinases. It is effective in patients resistant or intolerant to other TKIs [5].
Third	Ponatinib	Designed to overcome resistance caused by the T315I mutation, a particularly challenging mutation for other TKIs, ponatinib is highly effective but carries a risk of vascular complications [3].
	Asciminib	The drug is a first-in-class allosteric inhibitor that binds to the myristoyl pocket of BCR-ABL1, providing an alternative mechanism of action. It is particularly beneficial for patients who have exhausted previous TKI options due to resistance or intolerance [6].

**Table 2:** The distribution of tyrosine kinase inhibitors (TKIs) by treatment line within the RCLMC cohort. (Note: Percentages are based on patients evaluable for molecular response at each time point).

	Imatinib	Dasatinib	Nilotinib	Bosutinib	Ponatinib	Asciminib *	Total
^1^ L1	253	50	79	1	0	0	383
L2	9	36	61	8	5	0	119
L3	4	15	8	4	2	4	37
L4	0	0	0	6	2	1	9
L5	0	2	0	0	1	1	4
Total	266	103	148	19	10	6	

^1^ L: Line of treatment. * Asciminib was administered under compassionate treatment.

**Table 3:** Proportion of patients achieving specific molecular response milestones during first-line TKI therapy. (Note: Percentages refer to patients who discontinued TKI and had molecular follow-up data available at each time point).

First Line	BCR-ABL ≤ 1%	BCR-ABL ≤ 0.1%	MR4 and MR4.5
Month 3	58.25%	32.60%	
Month 6	88.67%	62.07%	
Month 12	95.05%	79.20%	50.50%
Month 18			29.20%
Month 24			69.60%

**Table 4:** Proportion of patients achieving specific molecular response milestones during second-line TKI therapy.

Second Line	BCR-ABL ≤ 1%	BCR-ABL ≤ 0.1%	MR4 and MR4.5
Month 3	87.50%	67.86%	
Month 6	96.70%	70%	
Month 12	98.50%	77.94%	51.80%
Month 18			50%
Month 24			52.94%

**Table 5:** Proportion of patients achieving specific molecular response milestones during third-line TKI therapy.

Third Line	CHR	CCyR	MMR	MR4	MR4.5
Month 12	60%	54.30%	40%	28.60%	14.30%

**Table 6:** The percentages of patients experiencing intolerance or suboptimal response/resistance in transitions between different treatment lines.

	Intolerance	Suboptimal Response or Resistance
L1 to L2	38.6%	61.6%
L2 to L3	29.73%	70.3%

**Table 7:** Association between early molecular response and subsequent deep molecular responses. Analysis restricted to first-line TKI therapy.

	*p* Value	OR	IC50
12 M	0.0269	3.29	1.15–9.42
18 M	0.8523	0.871	0.203–3.73
24 M	0.277	1.99	0.574–6.93

OR: Odds ratio; IC50: 50% confidence interval; M: months.

**Table 8:** Association between early molecular response and subsequent deep molecular responses. Analysis restricted to second-line TKI therapy.

	*p* Value	OR	IC50
12 M	0.178	7.93	0.388–162.07
18 M	0.151	9.26	1.44–192.7
24 M	0.175	8.13	0.394–167.9

OR: Odds ratio; IC50: 50% confidence interval; M: months.

## Data Availability

The datasets presented in this article are not readily available because the data are part of an ongoing study.

## Abbreviations

The following abbreviations are used in this manuscript:

CML-CP	Chronic Phase of Chronic Myeloid Leukemia
RCLMC	Canary Islands Registry of Chronic Myeloid Leukemia
qPCR	Quantitative Polymerase Chain Reaction
CML	Chronic Myeloid Leukemia
DMR	Deep Molecular Response
EMR	Early Molecular Response
TFR	Treatment-Free Remission
TKIs	Tyrosine Kinase Inhibitors
IS	International Scale
MR	Molecular Response Grade

## References

[1] Abruzzese E., Bocchia M., Trawinska M.M., Raspadori D., Bondanini F., Sicuranza A., Pacelli P., Re F., Cavalleri A., Farina M. (2023). Minimal residual disease detection at RNA and leukemic stem cell (LSC) levels: Comparison of RT-qPCR, d-PCR and CD26+ stem cell measurements in chronic myeloid leukemia (CML) patients in deep molecular response (DMR). Cancers.

[2] Stuckey R., López-Rodríguez J.F., Sánchez-Sosa S., Segura-Díaz A., Sánchez-Farías N., Bilbao-Sieyro C., Gómez-Casares M.T. (2020). Predictive indicators of successful tyrosine kinase inhibitor discontinuation in patients with chronic myeloid leukemia. World J. Clin. Oncol..

[3] Rinaldi I., Winston K. (2023). Chronic myeloid leukemia from pathophysiology to treatment-free remission: A narrative literature review. J. Blood Med..

[4] Shah N.P., García-Gutiérrez V., Jiménez-Velasco A., Larson S.M., Saussele S., Rea D., Mahon F.-X., Levy M.Y., Gómez-Casares M.T., Mauro M.J. (2023). Treatment-free remission after dasatinib in patients with chronic myeloid leukaemia in chronic phase with deep molecular response: Final 5-year analysis of DASFREE. Br. J. Haematol..

[5] Patterson S.D., Copland M. (2023). The bone marrow immune microenvironment in CML: Treatment responses, treatment-free remission, and therapeutic vulnerabilities. Curr. Hematol. Malig. Rep..

[6] Mauro M.J., Hughes T.P., Kim D.-W., Rea D., Cortes J.E., Hochhaus A., Sasaki K., Breccia M., Talpaz M., Ottmann O. (2023). Asciminib monotherapy in patients with CML-CP without BCR::ABL1 T315I mutations treated with at least two prior TKIs: 4-year phase 1 safety and efficacy results. Leukemia.

[7] Stuckey R., López Rodríguez J.F., Gómez-Casares M.T. (2022). Discontinuation of tyrosine kinase inhibitors in patients with chronic myeloid leukemia: A review of the biological factors associated with treatment-free remission. Curr. Oncol. Rep..

[8] White H.E., Salmon M., Albano F., Andersen C.S.A., Balabanov S., Balatzenko G., Barbany G., Cayuela J.-M., Cerveira N., Cochaux P. (2022). Standardization of molecular monitoring of CML: Results and recommendations from the European treatment and outcome study. Leukemia.

[9] Saugues S., Lambert C., Daguenet E., Johnson Ansah H., Turhan A., Huguet F., Guerci-Bresler A., Tchirkov A., Hamroun D., Hermet E. (2022). Real-world therapeutic response and tyrosine kinase inhibitor discontinuation in chronic phase-chronic myeloid leukemia: Data from the French observatory. Ann. Hematol..

[10] Costa A., Breccia M. (2024). How to improve treatment-free remission eligibility in chronic myeloid leukaemia?. Br. J. Haematol..

[11] Réa D., Boquimpani C., Mauro M.J., Minami Y., Allepuz A., Maheshwari V.K., D’Alessio D., Wu Y., Lawrance R., Narbutas S. (2023). Health-related quality of life of patients with resistant/intolerant chronic phase chronic myeloid leukemia treated with asciminib or bosutinib in the phase 3 ASCEMBL trial. Leukemia.

[12] Mu H., Zhu X., Jia H., Zhou L., Liu H. (2021). Combination therapies in chronic myeloid leukemia for potential treatment-free remission: Focus on leukemia stem cells and immune modulation. Front. Oncol..

[13] Hochhaus A., Baccarani M., Silver R.T., Schiffer C., Apperley J.F., Cervantes F., Clark R.E., Cortes J.E., Deininger M.W., Guilhot F. (2020). European LeukemiaNet 2020 recommendations for treating chronic myeloid leukemia. Leukemia.

[14] Réa D., Cayuela J.M. (2018). Treatment-free remission in patients with chronic myeloid leukemia. Int. J. Hematol..

[15] Hughes T.P., Clementino N.C.D., Fominykh M., Lipton J.H., Turkina A.G., Moiraghi E.B., Nicolini F.E., Takahashi N., Sacha T., Kim D.-W. (2021). Long-term treatment-free remission in patients with chronic myeloid leukemia after second-line nilotinib: ENESTop 5-year update. Leukemia.

[16] Hochhaus A., Masszi T., Giles F.J., Radich J.P., Ross D.M., Gómez Casares M.T., Hellmann A., Stentoft J., Conneally E., García-Gutiérrez V. (2017). Treatment-free remission following frontline nilotinib in patients with chronic myeloid leukemia in chronic phase: Results from the ENESTfreedom study. Leukemia.

[17] Efficace F., Mahon F.-X., Richter J., Piciocchi A., Cipriani M., Nicolini F.E., Mayer J., Zackova D., Janssen J.J.W.M., Panayiotidis P. (2021). Health-related quality of life and symptoms of chronic myeloid leukemia patients after discontinuation of tyrosine kinase inhibitors: Results from the EURO-SKI Trial. Leukemia.

[18] Iurlo A., Cattaneo D., Consonni D., Castagnetti F., Miggiano M.C., Binotto G., Bonifacio M., Rege-Cambrin G., Tiribelli M., Lunghi F. (2023). Treatment discontinuation following low-dose TKIs in 248 chronic myeloid leukemia patients: Updated results from a campus CML real-life study. Front. Pharmacol..

[19] Karg E., Baldow C., Zerjatke T., Clark R.E., Roeder I., Fassoni A.C., Glauche I. (2022). Modelling of immune response in chronic myeloid leukemia patients suggests potential for treatment reduction prior to cessation. Front. Oncol..

[20] Radich J.P., Hochhaus A., Masszi T., Hellmann A., Stentoft J., Gómez Casares M.T., García-Gutiérrez J.V., Conneally E., le Coutre P.D., Gattermann N. (2021). Treatment-free remission following frontline nilotinib in patients with chronic phase chronic myeloid leukemia: 5-year update of the ENESTfreedom trial. Leukemia.

[21] Jabbou E., Kantarjian H. (2020). Chronic myeloid leukemia: 2020 update on diagnosis, therapy and monitoring. Am. J. Hematol..

[22] Réa D., Mauro M.J., Boquimpani C., Minami Y., Lomaia E., Voloshin S., Turkina A., Kim D.-W., Apperley J.F., Abdo A. (2021). A phase 3, open-label, randomized study of asciminib, a STAMP inhibitor, vs bosutinib in CML after 2 or more prior TKIs. Blood.

[23] Claudiani S., Chughtai F., Khan A., Hayden C., Fernando F., Khorashad J., Orovboni V., Scandura G., Innes A., Apperley J.F. (2024). Long-term outcomes after upfront second-generation tyrosine kinase inhibitors for chronic myeloid leukemia: Managing intolerance and resistance. Leukemia.

[24] Harrington P., Radia D., de Lavallade H. (2020). What are the considerations for tyrosine kinase inhibitor discontinuation in chronic-phase chronic myeloid leukemia?. Expert Rev. Hematol..

[25] Hughes T.P., Saglio G., Kantarjian H.M., Guilhot F., Niederwieser D., Rosti G., Nakaseko C., De Souza C.A., Kalaycio M.E., Meier S. (2014). Early molecular response predicts outcomes in patients with chronic myeloid leukemia in chronic phase treated with frontline nilotinib or imatinib. Blood.

[26] Chen Y., Xu N., Yang Y., Liu Z., Xue M., Meng L., He Q., Chen C., Zeng Q., Zhu H. (2023). Quality-of-life, mental health, and perspective on TKI dose reduction as a prelude to discontinuation in chronic phase chronic myeloid leukemia. Cancer Med..

